# Evaluating the Utility of Fresh Tissue in Molecular Diagnostics of Colorectal Cancer

**DOI:** 10.3390/cancers17223709

**Published:** 2025-11-20

**Authors:** Tadeusz Kałużewski, Szymon Wcisło, Kinga Sałacińska, Łukasz Kępczyński, Izabela Kubiak, Magdalena Grabiec, Ewa Kalinka, Bogdan Kałużewski, Agnieszka Gach

**Affiliations:** 1 Department of Genetics, Polish Mother’s Memorial Hospital Research Institute, 93-338 Lodz, Poland; 2 Department of Thoracic, General and Oncological Surgery, Medical University of Lodz, 90-647 Lodz, Poland; 3 Laboratory of Medical Genetics, R&D Division, GENOS Sp. z o.o., 91-033 Lodz, Poland; 4 Department of Clinical Oncology, Medical University of Lodz, 92-231 Lodz, Poland; 5 Department of Oncology, Polish Mother’s Memorial Hospital Research Institute, 93-338 Lodz, Poland; ewa.kalinka@iczmp.edu.pl

**Keywords:** colorectal cancer, fresh tissue, molecular diagnostics, next-generation sequencing, oncogenic variants

## Abstract

**Background:** Molecular diagnostics has become a critical component of precision oncology in solid tumors, including colorectal cancer, yet the use of formalin-fixed, paraffin-embedded (FFPE) tissue often suffers from DNA degradation that compromises sequencing quality. This study aimed to evaluate the feasibility and effectiveness of using fresh, intraoperatively collected tumor tissue for next-generation sequencing-based molecular diagnostics in colorectal cancer. **Methods:** Tissue samples from 24 patients undergoing colorectal tumor resection were obtained based on macroscopic evaluation and tested with a custom gene panel. Sequencing metrics, mutation profiles, and correlations with clinical and pathological features were analyzed. **Results:** All samples yielded high-quality sequencing data. Oncogenic or likely oncogenic variants were detected in 21 out of 24 samples (87.5%), predominantly affecting genes frequently involved in colorectal cancer carcinogenesis, including *APC*, *TP53*, and *KRAS*. In three cases, no typical mutations were found despite visual confirmation of tumor tissue during surgery, which may be attributed to insufficient tumor cellularity or molecular alterations beyond the panel’s scope. **Conclusions:** The results support the use of fresh tissue as a high-quality source for molecular diagnostics, capable of reducing turnaround time and avoiding formalin-induced artifacts. However, the findings also highlight the diagnostic risk of relying solely on macroscopic tumor assessment without histological confirmation. Overall, fresh tissue-based testing represents a promising yet currently investigational approach that can enhance molecular diagnostics in colorectal cancer.

## Simple Summary

Colorectal cancer is a common oncological disease in which treatment decisions increasingly rely on detailed molecular testing of the tumor. In daily practice, such tests are usually performed on formalin-fixed, paraffin-embedded (FFPE) tissue, but fixation can damage DNA and reduce the quality of sequencing results. In this study, we explored whether small pieces of fresh tumor tissue collected directly during surgery could be used instead. We analyzed samples from 24 patients with colorectal cancer using a multigene next-generation sequencing panel. All fresh samples provided high-quality DNA and robust sequencing data, and cancer-driving mutations were identified in most tumors, mainly in *APC, TP53* and *KRAS* genes. Our findings indicate that fresh tissue is a promising source of high-quality material for molecular diagnostics and may help shorten turnaround time, but careful control of tumor cell content and further methodological refinement are needed before this approach can be safely implemented in routine practice.

## 1. Introduction

Colorectal cancer (CRC) remains one of the leading causes of cancer-related mortality worldwide and continues to represent a significant public health challenge [1]. In Poland, in 2021, CRC ranked as the third most common cancer among men and the fourth among women, with 10,009 and 7998 new cases, respectively. In terms of cancer-related deaths, it was the second leading cause among men (6570 cases) and the third among women (5022 cases) [2].

Advancements in diagnostic and therapeutic strategies have improved outcomes for CRC patients, with molecular testing emerging as a key component in expanding available treatment options [3]. Accurate and reliable molecular profiling of colorectal tumors is essential for guiding individualized treatment decisions and assessing prognosis [4]. Historically, molecular testing focused primarily on PCR-based detection of recurrent *KRAS*, *NRAS*, and *BRAF* mutations [5]. However, recent studies have continued to identify additional genes and molecular biomarkers relevant to colorectal cancer therapy [6]. As a result, there is a growing need to detect multiple concurrent alterations in tumor DNA using high-throughput technologies such as next-generation sequencing (NGS) [7,8]. However, high-throughput sequencing requires high-quality DNA to ensure reliable and accurate results [9,10]. This requirement highlights the importance of selecting appropriate sample types and preservation methods in clinical and research settings.

Formalin-fixed, paraffin-embedded (FFPE) tissue is currently the most commonly used sample type for molecular analysis [7]. Yet, fixation and long-term storage can lead to DNA cross-linking, fragmentation, and degradation. These factors may compromise both the quantity and quality of extracted DNA, potentially affecting the accuracy of mutation detection [11,12,13,14].

A key limitation associated with poor-quality DNA extracted from FFPE samples is the reduced presence of amplifiable DNA fragments of sufficient length [15]. This limitation negatively impacts the efficiency of library preparation, often leading to an increased proportion of PCR duplicates and uneven coverage across the sequenced regions, which are commonly observed characteristics of FFPE-derived material [16,17].

Particularly in the case of CRC, comparative studies emphasize that FFPE tissue stored for less than two years may serve as an acceptable source for mutation detection. However, various preanalytical and analytical factors—such as fixation time, storage conditions, and DNA integrity—can significantly impact the accuracy of results. From this perspective, FFPE should be used as an alternative only when fresh frozen tissue is not available, as discrepancies in mutation profiles may occur [18].

Nevertheless, an essential advantage of FFPE material is the ability to precisely assess tumor cell content through microscopic evaluation [19]. Consequently, current clinical guidelines recommend performing molecular testing only after prior or parallel histopathological verification by a pathologist, since the risk of obtaining a false-negative result due to the absence of tumor cells is considered higher than the risk associated with nucleic acid degradation during FFPE processing [20].

In cases of sufficiently large tumors, macroscopic evaluation during surgery may theoretically be adequate to identify tumor tissue suitable for molecular testing, which could then be conducted in parallel with histopathological assessment. This approach, applied selectively, may enable the acquisition of higher-quality material for genetic analysis and significantly shorten the time required to obtain molecular results. The present study aimed to evaluate this hypothesis using an in-house NGS-based multigene panel on fresh tissue samples collected during surgical resection from patients diagnosed with colorectal cancer.

## 2. Materials and Methods

### 2.1. Material Acquisition

Tissue samples were obtained from 24 patients diagnosed with colorectal adenocarcinoma during surgical procedures performed as part of routine therapeutic management. Small fragments of tissue, each measuring a few millimeters, were selected based on macroscopic examination, clearly indicative of tumor presence. Particular care was taken to ensure that sample collection did not interfere with the concurrent histopathological assessment, thereby preserving the diagnostic integrity of the specimens. The samples were immediately preserved in RNA Save solution (Biological Industries, Kibbutz Beit Haemek, Israel) and stored at 20 °C until nucleic acid isolation. All of the subjects included in the study had confirmed tumor cell content in sample parts that were subjected to parallel histopathological examinations.

### 2.2. DNA Isolation and Next-Generation Sequencing

Manual extraction of genomic DNA was performed using the Sherlock AX Kit (A&A Biotechnology, Gdansk, Poland) in accordance with the manufacturer’s instructions, following prior mechanical homogenization of the samples. Next-generation sequencing (NGS) was conducted using a 150 bp paired-end method on the MiniSeq platform (Illumina, San Diego, CA, USA). AmpliSeq DNA On-Demand libraries were prepared following the manufacturer’s instructions using the Mid Output Kit (Illumina, San Diego, CA, USA). A custom gene panel comprising 110 genes was designed using Illumina Design Studio software (Illumina, San Diego, CA, USA) to enable the sequencing of coding exons and adjacent non-coding regions. Panel content was based on a comprehensive review of the literature and databases such as PubMed and COSMIC. The following genes associated with colorectal cancer were included: *AKT1*, *APC*, *ARID1A*, *ARID1B*, *ATM*, *ATR*, *ATRX*, *AXIN2*, *BARD1*, *BIRC3*, *BMPR1A*, *BRAF*, *BRCA1*, *BRCA2*, *BRIP1*, *CACNA1D*, *CDC73*, *CDK12*, *CDKN1B*, *CDKN2A*, *CHEK2*, *CREBBP*, *CTNNB1*, *DICER1*, *EGFR*, *EPCAM*, *ERBB2*, *ERBB4*, *FANCC*, *FAT1*, *FAT4*, *FH*, *GDNF*, *GREM1*, *GRIN2A*, *HNF1A*, *HNF1B*, *HOXB13*, *KDR*, *KIF1B*, *KMT2A*, *KMT2C*, *KMT2D*, *KRAS*, *MAX*, *MC1R*, *MEN1*, *MET*, *MITF*, *MLH1*, *MLH3*, *MRE11*, *MSH2*, *MSH3*, *MSH6*, *MTOR*, *MUTYH*, *MYH11*, *NBN*, *NF1*, *NF2*, *NOD2*, *NTHL1*, *NRAS*, *NTRK3*, *PIK3CA*, *PMS2*, *POLD1*, *POLE*, *POT1*, *PPP2R1A*, *PRKAR1A*, *PRSS1*, *PTCH1*, *PTEN*, *PTPRB*, *PTPRC*, *RAD50*, *RAD51C*, *RAD51D*, *RB1*, *RET*, *RNF213*, *RNF43*, *RPS20*, *SDHA*, *SDHAF2*, *SDHB*, *SDHC*, *SDHD*, *SETBP1*, *SHH*, *SMAD4*, *SMARCA4*, *SMARCB1*, *SPEN*, *STK11*, *SUFU*, *TERT*, *TET1*, *TGFBR2*, *TMEM127*, *TP53*, *TRRAP*, *TSC1*, *TSC2*, *VHL*, *WT1*, *XRCC2*, *XRCC3*, *ZFHX3*.

### 2.3. Bioinformatics and Variant Classification

The identification of single-nucleotide variants (SNVs) as well as short insertions and deletions (delins) was performed using the GATK software suite (v4.6.0.0) [21], following the Best Practices recommendations [22,23]. Prior to variant calling, sequencing reads were aligned to the hg19 reference genome using BWA (v.0.7.17) [24], and converted to BAM format using SAMtools (v1.10). Quality metrics were generated with MultiQC (v1.22.2). Variant annotation was conducted using the Franklin platform (QIAGEN N.V., Hilden, Germany), and variant classification was performed according to the AMP/ACMG guidelines for pathogenicity and the ClinGen-CGC-VICC guidelines for oncogenicity. A graphical representation of the variant distribution was generated using Circos software (v0.69.9) [25].

## 3. Results

### 3.1. Patient Characteristics

A total of 24 patients were included in the study, comprising 6 men (mean age: 74.3 years) and 18 women (mean age: 70.0 years). The group comprised two female patients representing early-onset cases (age ≤ 50 years). The majority of patients were either overweight (*n* = 10) or obese (*n* = 7), while six patients had a normal body weight, and only one was underweight. Tumor samples were described in terms of size, pathological TNM classification (pTNM), and presence of metastases. Primary tumor location was rectum in 8/23 (34.8%) and colon in 15/23 (65.2%). Among colonic tumors, right-sided sites (cecum, ascending, transverse) accounted for 9/23 (39.1%), whereas left-sided sites (descending, sigmoid) accounted for 6/23 (26.1%). Histologic grade was predominantly G2 in 15/23 (65.2%) and G3 in 2/23 (8.7%). Grade was unreported/indeterminate in 6/23 (26.1%). Additionally, data on patients’ smoking history (pack-years) were collected. Detailed patient characteristics are presented in Table 1.

### 3.2. NGS Results

**Figure 1:**
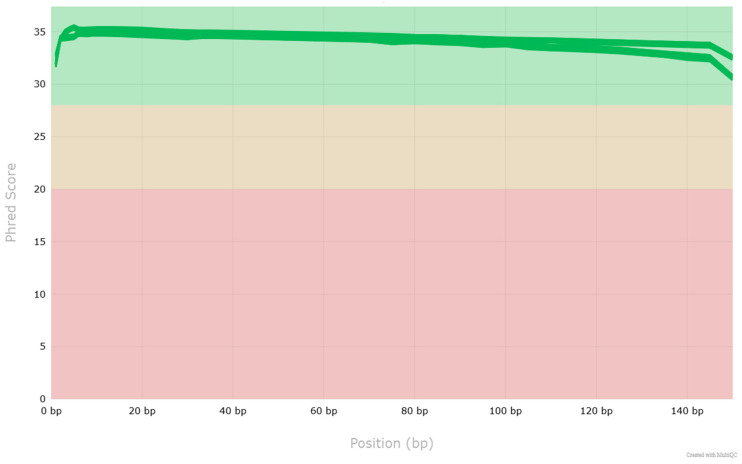
Mean quality scores as reported by FastQC. The x-axis represents the base position in the read, while the y-axis shows the mean Phred score for each position. The background colors correspond to the poor (red), medium(yellow) and satisfactory (green) quality. High-quality scores (above 30) indicate reliable base calls with low error probability. The plot reveals that the quality is highest at the beginning of the reads and gradually declines towards the end, which is a typical pattern in Illumina sequencing data. Despite the drop, the overall quality remains within acceptable ranges.

**Figure 2:**
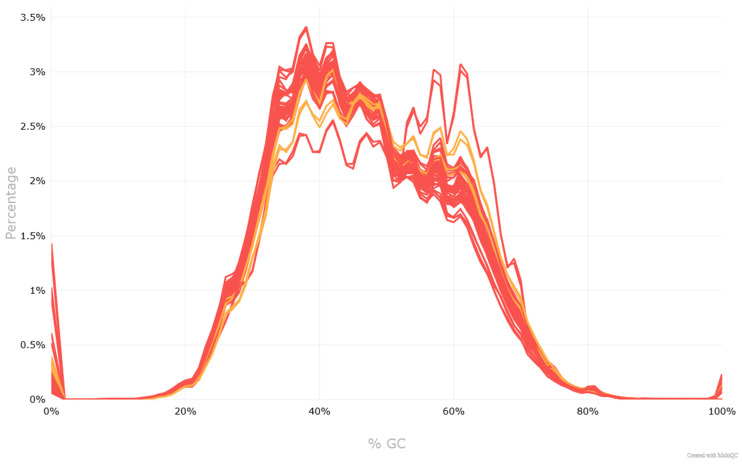
The x-axis represents the percentage of guanine and cytosine (GC) content per read, while the y-axis shows the percentage of reads falling into each GC content bin. The distribution generally follows a normal (bell-shaped) curve, which suggests a high-quality (red lines) or satisfactory (yellow lines) dataset. However, a slight shift in the peak positions between individual samples can be observed, which may reflect differences in GC content between samples or minor technical variation.

Next-generation sequencing was successfully performed for all samples. Quality assessment confirmed that the raw sequencing reads exhibited high quality across all 48 FASTQ files (Figure 1). Per-base sequence quality scores were consistently above Q30 for the majority of read positions, and no significant adapter contamination was detected. The GC content distribution matched the expected range of approximately 40–60% for human genomic DNA (Figure 2). Duplication levels were within acceptable limits for genomic DNA libraries. Mapping-based evaluation of the aligned BAM files revealed a mean sequencing depth of 101.56× across all tumor samples, with coverage values ranging from 56.29× to 129.89×. Coverage was uniformly distributed across the targeted genomic regions, with no pronounced dropout regions, confirming the suitability of the data for downstream analyses, including somatic mutation calling.

**Figure 3:**
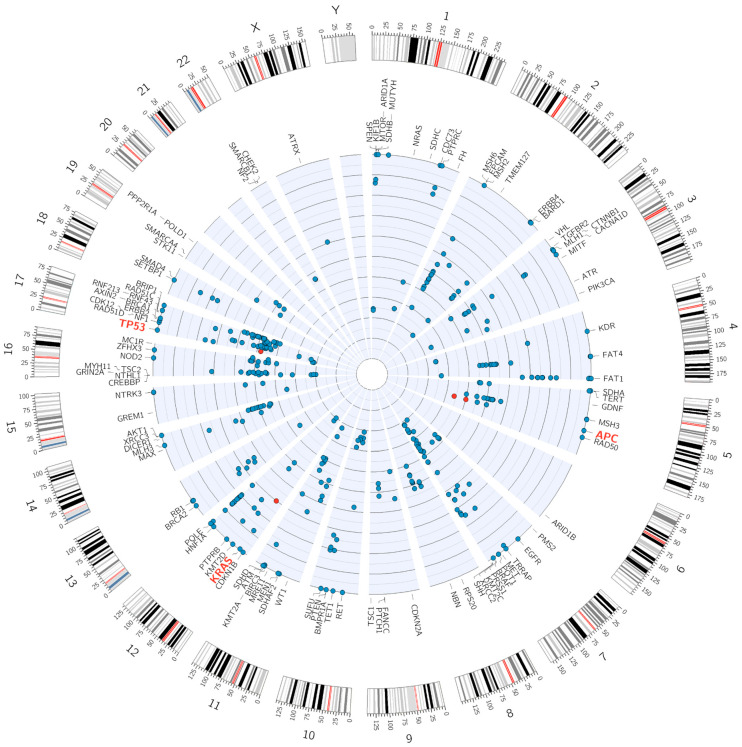
The Circos circular plot of genomic alterations in an example sample. The outermost ring represents ideograms of human chromosomes (1–22, X, Y). The inner radial plot displays genes analyzed in the panel, with each dot representing a detected variant. The position of each dot corresponds to its chromosomal location, while the radial distance from the center reflects the variant allele frequency (VAF) in the sample. Oncogenic variants within cancer-related genes (*TP53*, *APC*, *KRAS*) are highlighted in red.

**Figure 4:**
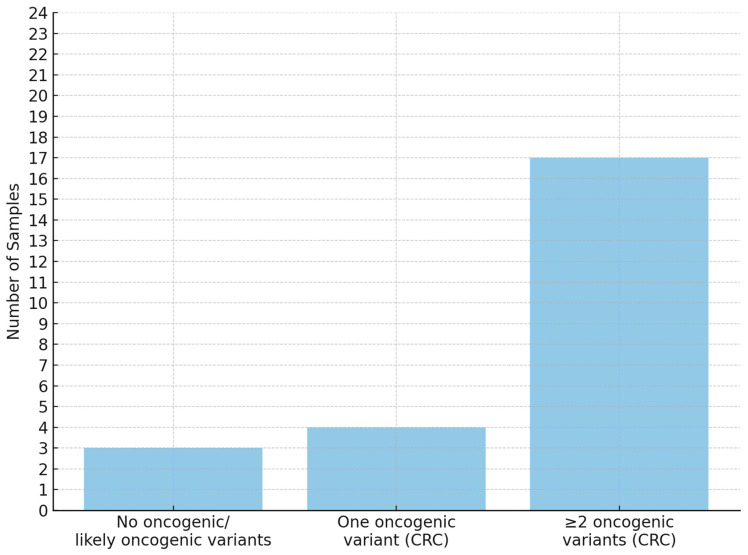
The bar chart presents the number of samples grouped by the presence of oncogenic or likely oncogenic variants typical for colorectal cancer. Seventeen samples carry two or more such variants, while four have one, and three have none.

The applied panel enabled the detection of a broad spectrum of point mutations as well as small insertions and deletions (Figure 3). In 18 out of 24 cases, oncogenic or potentially oncogenic variants were identified in genes associated with the conventional pathway (CIN—chromosomal instability pathway) of colorectal cancer development. The detected CIN-associated variants are presented in the following tables: *APC* (Table 2), *TP53* (Table 3), and *KRAS* (Table 4). In 3 out of 24 cases, oncogenic variants associated with the serrated or MSI (microsatellite instability) pathways were detected: the oncogenic *BRAF* c.1799T > A p.(Val600Glu) variant was found in two samples (classification criteria: OS1 + 4, OS3 + 4, OP1 + 1, OP4 + 1), while oncogenic *MSH6* NM_000179.3:c.3261dup p.(Phe1088Leufs*5) and likely oncogenic *TGFBR2* NM_003242.6:c.382_383del p.(Lys128Alafs*3) variants were found together in one sample (classification criteria: OVS1 + 8, OS1 + 4, OP4 + 1 and OVS1 + 8, OP4 + 1, respectively). In 3 of the 24 samples, no mutations typically associated with colorectal cancer were detected. The summary of oncogenic variant frequencies is presented in Figure 4.

### 3.3. Clinical Characteristics

Although investigating associations between patients’ clinical characteristics and detected genetic variants was not the primary objective of this study, exploratory analyses were conducted to expand the current understanding of variant relevance in a clinical context. The distribution of identified variants was analyzed in relation to tumor grade, size and localization, presence of metastases, patient age (<50 vs. ≥50 years), sex, body mass index (BMI), and duration of smoking history. No statistically significant correlations were observed within the examined cohort, which is likely attributable to the limited sample size and relatively low clinical heterogeneity of the study population. However, the applied panel allowed for the identification of a potentially germline variant in the *HOXB13* gene, NM_006361.6:c.251G>A p.(Gly84Glu), in a female patient. This specific variant is a well-characterized European founder mutation known to be associated with a significantly increased risk of prostate cancer, particularly in individuals of European ancestry [26]. Current evidence does not support an association between this variant and an increased risk of colorectal cancer or malignancies typically affecting women, such as breast or ovarian cancer [27]. The clinical relevance of this incidental finding in the context of a non-prostate cancer setting remains uncertain.

## 4. Discussion

The aim of this study was to evaluate the use of fresh tumor tissue for next-generation sequencing (NGS) in colorectal cancer (CRC) as a source of high-quality molecular data. All 24 fresh samples in our cohort were successfully sequenced with adequate depth and uniform coverage. Oncogenic or likely oncogenic variants were identified in the majority of cases (21/24, 87.5%). These results suggest that, when sufficient tumor content is present, intraoperative sampling of fresh tissue can provide DNA of excellent quality for mutation analysis and may potentially reduce turnaround time by enabling parallel processing with histopathology. Avoiding formalin fixation allows avoiding DNA crosslinking and degradation, which are known to introduce sequencing artifacts and reduce detectable variant yield, as previously described [18]. However, a critical limitation emerged in the few cases where no oncogenic variants were detected despite macroscopic tumor identification. In three patients, the used NGS panel did not reveal any typical CRC-associated mutations, raising concern for false-negative results. One possible explanation is an insufficient tumor cell content in the analyzed tissue fragment, meaning that the sample may have contained mostly non-neoplastic cells despite appearing tumorigenic on macroscopic evaluation. Another possibility is the absence of typical sequence mutations in the genes covered by the panel, which does not exclude other molecular alterations such as DNA methylation, genomic imbalances, or epigenetic instability [28]. Both of the above scenarios highlight the well-recognized risk of performing molecular testing on tissue that has not been histologically verified. The risk of a false-negative result might outweigh the advantages of improved DNA quality gained by immediate fresh tissue testing. Our observation of mutation-negative cases supports this concern, especially when considering the methodology used. Another notable finding from the study is that a subset of tumors harbored isolated *APC* mutations as the single driver alteration. *APC* is a tumor suppressor gene that acts as a gatekeeper in colorectal tumorigenesis, and it is well established that its mutations occur in more than 80% of sporadic CRCs very early in tumorgenesis [29,30,31]. Since these changes without additional findings may point to either biologically indolent tumor areas or limitations of the testing panel, the presence of an isolated *APC* truncating mutation in an apparently malignant tumor sample cannot be treated as a definitive molecular marker confirming the presence of CRC. These considerations naturally raise the question of how molecular diagnostic panels might be expanded. One strategy to increase diagnostic yield seems to be the use of broader DNA sequencing approaches, such as whole-exome or whole-genome sequencing, which could identify rare or novel driver mutations not included in targeted panels. Another complementary approach is to incorporate RNA-based profiling that could detect pathogenic gene fusions and aberrant splice variants that are undetectable through DNA sequencing.

The incidental finding in the study was the detection of a germline *HOXB13* p.(Gly84Glu) pathogenic variant in a female patient. It is a well-characterized hereditary mutation associated with increased prostate cancer risk. Therefore, its presence in a female patient with CRC is most likely a coincidental finding unrelated to her colorectal tumor. However, this case serves as an example of how broad tumor sequencing may incidentally uncover germline variants of potential clinical significance with implications for genetic counseling. As such, offering extended NGS panels should ideally include strategies for distinguishing somatic from germline variants, for example, by performing parallel sequencing of a matched peripheral blood sample. Determining the relevance of such findings seems to be a critical component of modern precision oncology.

## 5. Conclusions

In summary, our evaluation of fresh tissue for molecular diagnostics in CRC highlights both the promise and limitations of this approach. Performing NGS on unfixed tumor samples can provide highly accurate genomic information and potentially shorten the path to clinically actionable results. However, the applied methodology does not currently support routine clinical implementation for diagnostic purposes. The used custom-designed gene panel did not allow detection of many alterations currently known to be characteristic of tumor cells. Moreover, the cohort size limits the statistical significance and generalizability of our findings. Future strategies that enable the capture of the full spectrum of genomic alterations—including regulatory variants, deep intronic alterations, complex or large structural variants, epigenetic changes, and gene expression signatures—could refine the sensitivity of molecular diagnostics in CRC and better leverage the advantages of fresh tissue use. Recent advances in computational pathology, such as AI-based tumor cellularity assessment, may help address the limitations observed in our study. As recently demonstrated by Gertych et al. [32], artificial intelligence models trained on histology images can accurately predict the likelihood of successful molecular testing by quantifying tumor cell content prior to sequencing. Integrating such tools into routine workflows may significantly reduce the risk of false-negative results arising from inadequate tumor sampling. As precision oncology continues to advance, such approaches may become increasingly important in delivering timely and comprehensive genomic data to guide cancer patient management.

## Figures and Tables

**Table 1:** Detailed patient characteristics.

Sample	Sex	Age	BMI	Smoking (Pack-Year)	Localization	Grade	pTNM	Size (cm)	Lymph Nodes	Metastases
640	F	71	17.92	0	rectum	GX	T3N0M0	4.0 × 3.0 × 1.5	0/6	0
638	F	77	23.44	0	cecum	-	T3N0M0	5.0 × 4.0 × 1.5	0/18	0
631	F	69	26.85	20	rectum	G2	T3N0M0	5.5 × 6.0 × 0.7	0/22	0
625	M	79	26.12	0	rectum	G2	T3N2aM0	4.0 × 4.0 × 1.0	5/12	0
618	M	70	24.69	50	ascending colon	G3	T3N2aM0	6.5 × 4.5 × 2.0	5/15	0
616	F	79	25.39	6	ascending colon	G2	T2N2bM1a	5.5 × 6.0 × 3.0	9/20	liver
609	F	82	21.64	1	rectum	G2	T4bN2bM1a	6.0 × 3.0 × 2.1	7/13	spine
599	F	71	33.46	40	transverse colon	G2	T3N0M0	5.0 × 6.5 × 1.5	0/14	0
593	F	69	25.71	0	ascending colon	G3	T3N2aM0	6.0 × 3.5 × 1.5	4/7	0
577	M	77	25.35	30	rectum	G2	T2N0M0	5.0 × 7.5 × 2.0	0/28	0
570	F	78	25.97	0	cecum	-	T2N0M0	2.5 × 3.0 × 0.7	0/19	0
568	M	70	24.57	15	descending colon	G2	T3N1bM1a	2.5 × 3.5 × 2.5	3/13	liver
565	F	68	35.08	8	sigmoid colon	-	TisN0M0	3.0 × 2.5 × 2.0	0/0	0
560	F	63	31.25	0	ascending colon	G2	T3N0M0	4.5 × 4.5 × 2.5	0/14	0
555	F	82	24.22	0	sigmoid colon	G2	T2N0M0	7.0 × 4.5 × 0.8	0/13	0
554	M	77	26.20	20	ascending colon	-	T3N0M0	7.0 × 5.0 × 2.0	0/13	0
532	F	50	30.07	0	rectum	G2	T3N1bM0	4.5 × 3.0 × 1.0	2/19	0
526	F	33	22.15	0	rectum	G2	T2N0M0	2.0 × 2.0 × 1.0	0/13	0
507	M	73	37.02	0	sigmoid colon	G2	T3N0M0	3.2 × 3.0 × 1.0	0/14	0
504	F	74	30.86	10	ascending colon	G2	T4N0M0	8.0 × 4.0 × 1.0	0/14	0
505	F	81	27.11	0	rectum	G2	T3N2aM0	3.0 × 3.5 × 1.0	4/13	0
493	F	73	26.14	0	sigmoid colon	G2	T3N0M0	3.7 × 4.0 × 1.0	0/8	0
486	F	75	31.65	0	sigmoid colon	-	T2N0M0	3.5 × 2.0 × 1.0	0/13	0
484	F	65	27.64	0	rectum	G2	T2N0M0	6.2 × 5.0 × 2.0	0/16	0

**Table 2:** Oncogenic and likely oncogenic *APC* (NM_000038.6) variants identified in the study.

Sample	Nucleotide Variant	Predicted Protein Variant	ClinGen-CGC-VICC Pathogenicity	Classification Criteria
618	c.543_546del	p.(Thr182Ilefs*2)	Likely Oncogenic	OVS1 + 8, OP4 + 1
532	c.646C>T	p.(Arg216*)	Oncogenic	OVS1 + 8, OS1 + 4, OS3 + 4, OP4 + 1
505	c.847C>T	p.(Arg283*)	Oncogenic	OVS1 + 8, OS1 + 4, OS3 + 4, OP4 + 1
526	c.1495C>T	p.(Arg499*)	Oncogenic	OVS1 + 8, OS1 + 4, OS3 + 4, OP4 + 1
577	c.1690C>T	p.(Arg564*)	Oncogenic	OVS1 + 8, OS3 + 4, OP4 + 1
631	c.2336del	p.(Leu779*)	Likely Oncogenic	OVS1 + 8, OP4 + 1
599	c.2413C>T	p.(Arg805*)	Oncogenic	OVS1 + 8, OS1 + 4, OS3 + 4, OP4 + 1
484	c.2804dup	p.(Tyr935*)	Oncogenic	OVS1 + 8, OM3 + 2, OP4 + 1
616	c.2928_2929del	p.(Gly977Serfs*7)	Likely Oncogenic	OVS1 + 8, OP4 + 1
555	c.3340C>T	p.(Arg1114*)	Oncogenic	OVS1 + 8, OS3 + 4, OP4 + 1
486	c.3454C>T	p.(Gln1152*)	Likely Oncogenic	OVS1 + 8, OP4 + 1
625	c.3852del	p.(Asp1285Metfs*3)	Likely Oncogenic	OVS1 + 8, OP4 + 1
532	c.3859del	p.(Ile1287*)	Oncogenic	OVS1 + 8, OM3 + 2, OP4 + 1
555	c.3907C>T	p.(Gln1303*)	Oncogenic	OVS1 + 8, OS3 + 4, OP4 + 1
609, 526, 609	c.3927_3931del	p.(Glu1309Aspfs*4)	Oncogenic	OVS1 + 8, OS1 + 4, OS3 + 4, OP4 + 1
493, 593	c.4033G>T	p.(Glu1345*)	Oncogenic	OVS1 + 8, OS3 + 4, OP4 + 1
565	c.4129_4130del	p.(Val1377Serfs*8)	Likely Oncogenic	OVS1 + 8, OP4 + 1
505	c.4135G>T	p.(Glu1379*)	Oncogenic	OVS1 + 8, OS3 + 4, OP4 + 1
484	c.4391_4394del	p.(Glu1464Valfs*8)	Oncogenic	OVS1 + 8, OS1 + 4, OS3 + 4, OP4 + 1
507	c.4473dup	p.(Ala1492Cysfs*22)	Likely Oncogenic	OVS1 + 8, OP4 + 1
616	c.4666dup	p.(Thr1556Asnfs*3)	Oncogenic	OVS1 + 8, OS3 + 4, OP4 + 1
554	c.4741del	p.(Ser1581Leufs*69)	Oncogenic	OVS1 + 8, OM3 + 2, OP4 + 1

**Table 3:** Oncogenic and likely oncogenic *TP53* (NM_000546.6) variants identified in the study.

Sample	Nucleotide Variant	Predicted Protein Variant	ClinGen-CGC-VICC Pathogenicity	Classification Criteria
493, 593	c.378C>A	p.(Tyr126*)	Oncogenic	OVS1 + 8, OS1 + 4, OM1 + 2, OP4 + 1
631	c.389T>C	p.(Leu130Pro)	Oncogenic	OS2 + 4, OM1 + 2, OP1 + 1, OP3 + 1, OP4 + 1
577	c.396G>C	p.(Lys132Asn)	Oncogenic	OS1 + 4, OS2 + 4, OS3 + 4, OP1 + 1, OP4 + 1
526	c.475G>C	p.(Ala159Pro)	Oncogenic	OS2 + 4, OS3 + 4, OP1 + 1, OP4 + 1
554	c.487T>C	p.(Tyr163His)	Oncogenic	OS2 + 4, OS3 + 4, OP1 + 1, OP4 + 1
618	c.524G>A	p.(Arg175His)	Oncogenic	OS1 + 4, OS2 + 4, OS3 + 4, OP1 + 1, OP4 + 1
599	c.527G>T	p.(Cys176Phe)	Oncogenic	OS1 + 4, OS2 + 4, OS3 + 4, OP1 + 1, OP4 + 1
505, 532	c.743G>A	p.(Arg248Gln)	Oncogenic	OS1 + 4, OS2 + 4, OS3 + 4, OP1 + 1, OP4 + 1
486	c.809T>G	p.(Phe270Cys)	Likely Oncogenic	OS2 + 4, OM3 + 4, OP1 + 1, OP4 + 1
616	c.818G>A	p.(Arg273His)	Oncogenic	OS1 + 4, OS2 + 4, OS3 + 4, OP1 + 1, OP4 + 1
507	c.844C>T	p.(Arg282Trp)	Oncogenic	OS1 + 4, OS2 + 4, OS3 + 4, OP1 + 1, OP4 + 1
625	c.1024C>T	p.(Arg342*)	Oncogenic	OVS1 + 8, OS1 + 4, OP4 + 1

**Table 4:** Oncogenic and likely oncogenic *KRAS* (NM_004985.5) variants identified in the study.

Sample	Nucleotide Variant	Predicted Protein Variant	ClinGen-CGC-VICC Pathogenicity	Classification Criteria
505	c.35G>T	p.Gly12Val	Oncogenic	OS1 + 4, OS3 + 4, OP1 + 1, OP4 + 1
507, 526, 616, 599	c.35G>A	p.Gly12Asp	Oncogenic	OS1 + 4, OS3 + 4, OP1 + 1, OP4 + 1
484, 631	c.38G>A	p.Gly13Asp	Oncogenic	OS1 + 4, OS3 + 4, OP1 + 1, OP4 + 1

## Data Availability

The data and materials used in this study are available from the corresponding author upon reasonable request.
